# Improvement of Endothelial Dysfunction of Berberine in Atherosclerotic Mice and Mechanism Exploring through TMT-Based Proteomics

**DOI:** 10.1155/2020/8683404

**Published:** 2020-05-31

**Authors:** Wangxiao Tan, Yu Wang, Kaiyue Wang, Siwei Wang, Jinghua Liu, Xiaoyan Qin, Yongna Dai, Xiaoying Wang, Xiumei Gao

**Affiliations:** State Key Laboratory of Modern Chinese Medicine, Tianjin University of Traditional Chinese Medicine, Tianjin 301617, China

## Abstract

Atherosclerosis is a multifactorial vascular disease triggered by disordered lipid metabolism, characterized by chronic inflammatory injury, and initiated by endothelial dysfunction. Berberine is the main active alkaloid of the herbal medicine *Coptidis Rhizoma* (Huanglian). Notably, berberine has been shown to have beneficial effects against atherosclerosis. However, the mechanisms of berberine in preventing atherosclerosis are still unclear. This study is aimed at investigating the effects and mechanisms of berberine in protecting the aorta and ameliorating atherosclerosis in apolipoprotein E-deficient (ApoE^−/−^) mice. Here, we demonstrated that berberine reduced serum lipid levels, antagonized hepatic lipid accumulation, improved intima-media thickening, and alleviated atherosclerotic lesions in ApoE^−/−^ mice fed a western-type diet for 12 weeks. Meanwhile, berberine reduced aortic reactive oxygen species (ROS) generation and reduced the serum levels of malondialdehyde (MDA), oxidized low-density lipoprotein (ox-LDL), and interleukin-6 (IL-6). In aortic ring assay, berberine restored aortic endothelium-dependent vasodilatation in vivo and in vitro. Furthermore, 4,956 proteins were identified by proteomic analysis, and 199 differentially expressed proteins regulated by berberine were found to be involved in many biological pathways, such as mitochondrial dysfunction, fatty acid *β*-oxidation I, and FXR/RXR activation. Summarily, these data suggested that berberine ameliorates endothelial dysfunction and protects against atherosclerosis, and thus may be a promising therapeutic candidate for atherosclerosis.

## 1. Introduction

Atherosclerosis, a progressive chronic inflammatory disease characterized by lipid deposition, smooth muscle cell proliferation, mononuclear cell infiltration, calcification, and plaque formation, usually occurs in large and medium-sized arteries and is the main cause and pathological basis of cardiovascular disease, which is the leading cause of human death, accounting for 31.5% of all deaths worldwide [1–3]. Many risk factors affect atherosclerosis, including gender, smoking, obesity, diabetes, and hypertension, and interaction of multiple risk factors leads to the occurrence and development of atherosclerosis [4]. Although the pathogenesis of atherosclerosis is still not completely clear, it is known to involve disorder of lipid metabolism, endothelial dysfunction, inflammation, and oxidative stress. Endothelial dysfunction is an early key step in the development of atherosclerosis, initiates the progression of atherosclerosis, and is involved in plaque formation. Moreover, the status of endothelial function can potentially reflect the propensity of atherosclerosis occurrence [5, 6]. Oxidized low-density lipoprotein (ox-LDL) is a major contributor to endothelial dysfunction in atherosclerosis; its formatting is triggered by enhanced blood lipid levels and oxidative stress, and it is deposited in the vascular endothelium [7]. Inflammation is also involved in endothelial dysfunction, with the activation of inflammatory cells and molecules, including adhesion molecules and chemokines [8]. When the endothelium is injured, it stimulates the expression of inflammatory mediators, including vascular cell adhesion molecule-1 (VCAM-1), intercellular adhesion molecule-1 (ICAM-1), interleukin 6 (IL-6), and tumor necrosis factor *α* (TNF-*α*), which activate oxidative stress and the inflammatory response and induce vascular smooth muscle cell proliferation and foam cell formation, leading to plaque formation and eventually atherosclerotic lesions [9, 10].

Berberine, an isoquinoline alkaloid, is the main active ingredient of the herbal medicine *Coptidis Rhizoma* (Huanglian), which has traditionally been used for treating diarrhoea in China and other Asian countries. Recently, increasing in vitro and in vivo studies have found that berberine has significant pharmacological effects on the cardiovascular system, including lowering serum lipid levels [11], lowering blood sugar [12], protecting the vascular endothelium [13], and anti-inflammatory [14] and antioxidant [15] effects. Notably, berberine has shown beneficial effects against atherosclerosis. Berberine was found to suppress atherogenesis through activation of AMPK-dependent UCP2 expression [16] and stabilize atherosclerosis plaques in hyperhomocysteinemia mice via activation of peroxisome proliferator-activated receptor gamma (PPAR*γ*) [17]. Moreover, the atheroprotective activities of berberine could be linked to inhibition of cholesterol accumulation in macrophages [18]. Nevertheless, there is still a lack of understanding of the underlying mechanisms by which berberine protects the aorta and ameliorates atherosclerosis.

With the recent advances in mass spectrometry (MS) technology, MS-based proteomics can rapidly identify and accurately quantify the proteomes of various samples within a single experiment and has been widely used for target identification and mechanistic studies [19, 20]. As one proteomics method, Tandem Mass Tag- (TMT-) based liquid chromatography-tandem mass spectrometry (LC-MS/MS) analysis can identify and quantify proteins in up to 10 biological samples simultaneously [21]. This method has been an essential tool in quantitative proteomics and can provide an overview of treatment mechanisms.

Here, we explored the protective effects and potential mechanism of berberine in atherosclerotic aorta in vivo and in vitro using apolipoprotein E-deficient (ApoE^−/−^) mice. ApoE^−/−^ mice are a widely used murine model for atherosclerosis [22]. We investigated the lipids, aortic histology, endothelial function, oxidative stress, and inflammatory markers in ApoE^−/−^ mice fed a high-fat diet. Furthermore, TMT-based LC-MS/MS technology and bioinformatics analysis were applied to analyse how berberine protects against atherosclerosis by altering the aorta proteome.

## 2. Materials and Methods

### 2.1. Materials

Berberine was purchased from Chengdu Mansite Pharmaceutical Co., Ltd. (A0151, Chengdu, China). Atorvastatin was purchased from Pfizer Pharmaceutical Co., Ltd. (Dalian, China). The western-type diet was purchased from Beijing HFK Bioscience (Beijing, China, Permission Number: SCXK (Jing) 2014-0008). Isoflurane was purchased Abbott Laboratories (Shanghai, China). NaCl, KCl, MgSO_4_·7H_2_O, KH_2_PO_4_, CaCl_2_, NaHCO_3_, and glucose in this article were purchased from Tianjin Kemiou Chemical Reagent Co., Ltd. (Tianjin, China). Acetonitrile was purchased from Merck (Germany). Norepinephrine (NE), acetylcholine (Ach), sodium nitroprusside (SNP), and formic acid were purchased from Sigma-Aldrich (St. Louis, MO, USA). The endothelial nitric oxide synthase (eNOS) antibody (RT1203) was purchased from HuaBio (Hangzhou, China). The CD31 antibody (550274) was purchased from BD (USA). Endothelin 1 (ET-1) (ab117757), glycerol-3-phospate dehydrogenase 2 (GPD2) (ab188585), paraoxonase 1 (PON1) (ab24261), and apolipoprotein A I (APOA1) (ab20453) antibodies were purchased from Abcam (Cambridge, UK). Glyceraldehyde-3-phosphate dehydrogenase (GAPDH) antibody (CW0100A), goat anti-mouse IgG (CW0102) and goat anti-rabbit IgG (CW0103) were purchased from CoWin Biosciences (Beijing, China). Trypsin was purchased from Thermo Fisher Scientific (NJ, USA).

### 2.2. Animals and Treatment

Male wild-type C57BL/6 mice (6 to 8 weeks old) and ApoE^−/−^ mice (6 to 8 weeks old) on a C57BL/6 genetic background were obtained from Beijing Vital River Laboratory Animal Technology Co., Ltd. (Beijing, China), and the permission numbers are SCXK (Jing) 2016-0006 and SCXK (Jing) 2016-0011. All animals were housed in standard cages (≤5 mice/cage) with clean sawdust bedding under specific-pathogen-free environmental conditions (20-25°C, 40-60% relative humidity, 12 h light/dark cycle) and had free access to water and food. All procedures were strictly in accordance with the Guidelines for the Care and Use of Laboratory Animals issued by the Ministry of Science and Technology of China and approved by the Laboratory Animal Ethics Committee of Tianjin University of Traditional Chinese Medicine (approval number: TCM-LAEC2016014).

After being acclimatized to the housing environment for one week, fifty two ApoE^−/−^ mice fed a western-type diet containing 21% fat and 0.15% cholesterol were randomly divided into four groups: the vehicle group (ApoE^−/−^, *n* = 15), the atorvastatin group (ATO, 2.6 mg·kg^−1^, *n* = 15), the berberine 78 mg·kg^−1^ group (BBR-78 mg·kg^−1^, *n* = 7), and the berberine 156 mg·kg^−1^ group (BBR-156 mg·kg^−1^, *n* = 15). The wild-type C57BL/6 mice fed a normal chow diet were used as the control group (WT, *n* = 15). The vehicle and control mice were treated with physiological saline. All the mice were administered treatments by oral gavage for 12 weeks.

### 2.3. Ultrasonography Analysis

Ultrasonography was performed after 12 weeks of administration using a Vevo 2100 ultra-high-resolution small animal ultrasound imaging system (VisualSonics, Toronto, Canada) equipped with an MS550D transducer operating at 40 MHz. Mice were anaesthetized with 1.5-2.0% inhalant isoflurane in 100% oxygen. After the hair was carefully removed from the chest and neck, the mice were laid on a heated platform in the supine position. First, the parasternal long-axis view was employed to visualize the left ventricle and ascending aorta, and M-mode and PW Doppler mode images were acquired. Then, the scan head was moved to the neck, where the right common carotid artery was visualized using a long-axis view. On B-mode, ultrasound images of the right common carotid artery were obtained. All the cine loops and frames were recorded and stored digitally for image analysis using an off-line workstation Vevo LAB 2.1.0 (VisualSonics, Toronto, Canada).

At the end of the experiment, mice were sacrificed, followed by blood sample, liver, aorta, and heart collection.

### 2.4. Organ Chamber

The organ chamber experiments were carried out as described previously [23]. Mice were sacrificed by decapitation, and the thoracic aorta was isolated and immediately immersed in oxygenated ice-cold Krebs-Henseleit (K-H) solution (composition in mM: NaCl 118, KCl 4.75, MgSO_4_·7H_2_O 1.2, KH_2_PO_4_ 1.2, CaCl_2_ 2.5, NaHCO_3_ 25, and glucose 11; pH 7.3-7.4). After careful removal of the adhering perivascular tissues, 2-3 mm rings were cautiously cut. The aortic rings were suspended in an organ bath containing 10 mL K-H solution maintained at 37°C and bubbled with 95% O_2_ and 5% CO_2_ between two parallel stainless hooks. The isometric tension during the experiments was measured and recorded by a computer-assisted data acquisition system (PowerLab/4SP; ADInstruments Pty Ltd., Australia). Each ring was stretched progressively to a baseline tension of 0.8 g and allowed to equilibrate for 60 min prior to the experiment. After the equilibration, the rings were initially contracted with 60 mM KCl to elicit their contractile response, and then, they were rinsed with K-H solution three times to restore tension to the basal level. Subsequently, NE (10^−6^ M) was used to precontract the rings. At the plateau of contraction, ACh (10^−9^-10^−4^ M) or SNP (10^−9^-10^−5^ M) was added cumulatively into the organ bath to induce endothelium-dependent or endothelium-independent relaxation.

For ex vivo experiments, the rings were preincubated with berberine (10^−7^, 5 × 10^−7^, 10^−6^ M) for 30 min. Then, the rings were washed with K-H solution, after which ACh-induced endothelium-dependent relaxation and SNP-induced endothelium-independent relaxation were assayed separately.

### 2.5. Determination of Serum Lipids, Ox-LDL, MDA, and IL-6

Serum was prepared from blood samples. Triglyceride (TG), total cholesterol (TC), high-density lipoprotein cholesterol (HDL-C), low-density lipoprotein cholesterol (LDL-C), and free fatty acids (FFA) were measured using biochemical kits (Nanjing Jiancheng Bioengineering Institute, Nanjing, China); ox-LDL and malondialdehyde (MDA) were determined with ELISA kits (Cloud-Clone Corp., Wuhan, China); and IL-6 was determined with ELISA kits (Nanjing Jiancheng Bioengineering Institute, Nanjing, China). All the procedures were performed according to the manufacturer's instructions.

### 2.6. Hepatic Histology

After being photographed and weighed, a piece of liver was fixed in 10% neutral formalin, and the remaining portion was frozen in liquid nitrogen and preserved at -80°C. Liver tissues fixed in formalin were dehydrated with gradient ethanol and embedded in paraffin. The paraffin-embedded specimens were sliced into 4 *μ*m thick serial sections, which were stained with haematoxylin-eosin (H&E, Wuhan Servicebio Biological Technology Co., Ltd., Wuhan, China) in accordance with the standard protocol. To observe lipid accumulation in the liver, the frozen tissues were embedded in optimum cutting temperature compound and cut into 6 *μ*m thick cryosections. The sections were stained with Oil Red O (Sigma-Aldrich, St. Louis, MO, USA). Images of all sections were obtained with a light microscope (Eclipse CI, Nikon Corporation, Tokyo, Japan).

### 2.7. Assessment of Aortic Atherosclerosis

Mice were perfused with 0.9% ice-cold saline through the left ventricle after blood collection. The hearts containing the aortic root were removed and fixed in 10% neutral formalin for paraffin sectioning or frozen in liquid nitrogen for cryosectioning. H&E, Oil Red O, and Verhoeff-Van Gieson (VVG, Wuhan Servicebio Biological Technology Co., Ltd., Wuhan, China) staining were performed. All the images were captured with a light microscope. Quantification of positive Oil Red O staining was performed using Image-Pro Plus 6.0 software (Media Cybernetics Inc., USA).

### 2.8. Immunofluorescence and TUNEL Staining

To evaluate vascular endothelial function, the expression of eNOS and ET-1 in the aortic root was determined by immunofluorescence staining. CD31 and terminal deoxynucleotidyl transferase-mediated dUTP nick-end labelling (TUNEL) double immunofluorescence staining was performed to measure endothelial apoptosis. The cryosections of aortic sinuses were permeabilized with 0.2-0.5% Triton X-100 for 10 min. The primary antibodies anti-eNOS, anti-ET-1, and anti-CD31 were diluted (1 : 200) and incubated overnight at 4°C with the sections. After washing in PBS (3 × 3 min), the sections were incubated with secondary antibodies (donkey anti-rat IgG (ab150154, Abcam) and donkey anti-rabbit IgG (ab150073, Abcam, Cambridge, UK), 1 : 200 dilution) for 1 h at room temperature. Thereafter, TUNEL costaining was performed using an In Situ Cell Death Detection Kit (11684795910, Roche, Germany) in accordance with the manufacturer's protocol for double fluorescence staining, and the cell nuclei were counterstained with 4′,6-diamidino-2-phenylindole (DAPI) (H1200, Vector Laboratories, CA, USA) for all sections. Fluorescence staining was visualized under a fluorescence microscope (DFC500, Leica, Wetzlar, Germany).

### 2.9. Detection of Aortic ROS Production

The frozen cross sections of the aortic root were incubated with 10 *μ*M dihydroethidium (DHE) (S0063, Beyotime Institute of Biotechnology, Jiangsu, China) in a dark humidified chamber at 37°C for 30 min. *In situ* reactive oxygen species (ROS) generation labelled with red fluorescence was visualized and photographed using a fluorescence microscope.

### 2.10. TMT-Based Proteomics Analysis

#### 2.10.1. Protein Preparation and TMT Labelling

Aortic tissues were lysed in lysis buffer at 70 Hz for 120 s. The lysed tissue samples were centrifuged, and the supernatant was collected. The protein concentration was quantified using the Bradford method [24]. After quantification, the protein extracted from each sample was digested with 0.5 *μ*g·*μ*L^−1^ trypsin solution (Thermo Fisher Scientific, NJ, USA). Then, TMT labelling was performed with a 10-plex TMT kit (Thermo Fisher Scientific, NJ, USA). Peptides in each group were labelled with different TMT labels: three biological repeats of the wild-type mice group were labelled TMT-127N, TMT-127C, and TMT-128N; three biological repeats of the ApoE^−/−^ group were labelled TMT-128C, TMT-129N, and TMT-129C; and three biological repeats of the berberine 156 mg·kg^−1^ group were labelled TMT-130N, TMT-130C, and TMT-131. All the labelled samples were mixed, vacuum dried, and stored at -20°C for further analysis.

#### 2.10.2. High pH Reversed-Phase Fractionation and LC-MS/MS Analysis

The TMT-labelled peptides were dissolved in 100 *μ*L buffer A (98% double-distilled water, 2% acetonitrile, pH 10) and fractionated via high pH reversed-phase fractionation chromatography with a RIGOL L-3000 HPLC System (Beijing RIGOL Technologies Inc., Beijing, China). Fractions were collected every 1.75 min in 45 tubes and then dried and combined into 10 tubes for further LC-MS/MS analysis.

LC-MS/MS analysis was performed with an EASY-nLC 1000 System (Nano HPLC, Thermo Fisher Scientific, USA) coupled online to a Q Exactive Mass Spectrometer with an EASY-Spray Ion Source (Thermo Fisher Scientific, USA). The samples were loaded onto an Acclaim PepMap 100 precolumn (2 cm × 100 *μ*m, 5 *μ*m, C18, Thermo Fisher Scientific, USA). Peptide separation was conducted using an EASY-Spray column (12 cm × 75 *μ*m, 3 *μ*m, C18, Thermo Fisher Scientific, USA) with buffer A (100% ultrapure water and 0.1% formic acid) and buffer B (100% acetonitrile and 0.1% formic acid) at a flow rate of 350 nL·min^−1^. The eluted peptides were analysed using the Q Exactive online system. MS data acquisition was performed using a data-dependent top 20 method.

#### 2.10.3. Protein Identification and Quantitative Analysis

The LC-MS/MS raw data were searched against the mouse FASTA database from UniProt (Uniprot_mouse_54106_20170101.fasta) using Proteome Discoverer version 2.1 (Thermo Fisher Scientific, USA). The false discovery rate (FDR) was set as <0.01. Detection of at least one unique peptide per protein was set as the requirement for protein identification. The protein quantitative analysis was based on reporter ion peak intensity. Differentially expressed proteins were identified through ratio-fold change as well as *P* value calculated with a *t*-test. The up- and downregulation thresholds were set at an average ratio − fold change > 1.2 with a *P* value < 0.05 and an average ratio − fold change < 0.83 with a *P* value < 0.05; the proteins that satisfied the set thresholds were collected as “Differentially expressed proteins” for further bioinformatics analysis.

#### 2.10.4. Bioinformatics Analysis

Functional annotations of differentially expressed proteins were performed through Gene Ontology (GO) enrichment analysis (http://www.geneontology.org/). In addition, Ingenuity Pathway Analysis (IPA) software (QIAGEN, CA, USA, http://www.ingenuity.com) was used to analyse canonical pathways, diseases and functions, and biological networks associated with the lists of differentially expressed proteins. The dataset containing differentially expressed proteins and corresponding expression values was uploaded into the IPA, and then, IPA “Core Analysis” was performed.

#### 2.10.5. Western Blotting

The protein from aortic tissues was extracted and quantified as described in Section 2.10.1. Equal amounts (20 *μ*g) of total protein from each sample were separated by 10% sodium dodecyl sulfate-polyacrylamide gel electrophoresis (SDS-PAGE), followed by transfer onto polyvinylidene fluoride (PVDF) membranes (Millipore, MA, USA). Then, the membranes were blocked with 5% skim milk at 37°C for 2 h and incubated with anti-GPD2 (diluted 1 : 1000), anti-PON1 (diluted 1 : 1000), anti-APOA1 (diluted 1 : 1000), and anti-GAPDH (diluted 1 : 1000) antibodies overnight at 4°C. The membranes were washed with 0.5% Tween-20/PBS (PBST) 4 × 5 min and incubated with horseradish peroxidase- (HRP-) conjugated goat anti-mouse or goat anti-rabbit secondary antibody (1 : 2000) at room temperature for 1 h. After four washes with PBST, proteins were visualized using ECL Western Blotting Substrate (32106, Thermo Fisher Scientific, NJ, USA) and exposed to X-ray film (6535876, Eastman Kodak Company, NY, USA). The protein expression level was defined as the grey value quantified with ImageJ software (National Institutes of Health, USA) and normalized to GAPDH expression.

### 2.11. Statistical Analysis

All the experimental data are expressed as the mean ± SEM. Statistical analyses were performed with SPSS software (version 17.0, SPSS Inc., Chicago, IL, USA). One-way ANOVA with Tukey's post hoc test or a Kruskal-Wallis test followed by Dunn's multiple comparison was used for comparisons among multiple groups. Student's *t*-test was used for comparisons of two groups. Differences with *P* < 0.05 were considered statistically significant. GraphPad Prism 5.0 (GraphPad Software Inc., La Jolla, CA, US) was used for the graphical work.

## 3. Results

### 3.1. Berberine Reduced Serum FFA and TG Levels and Antagonized Hepatic Lipid Accumulation

**Figure 1:**
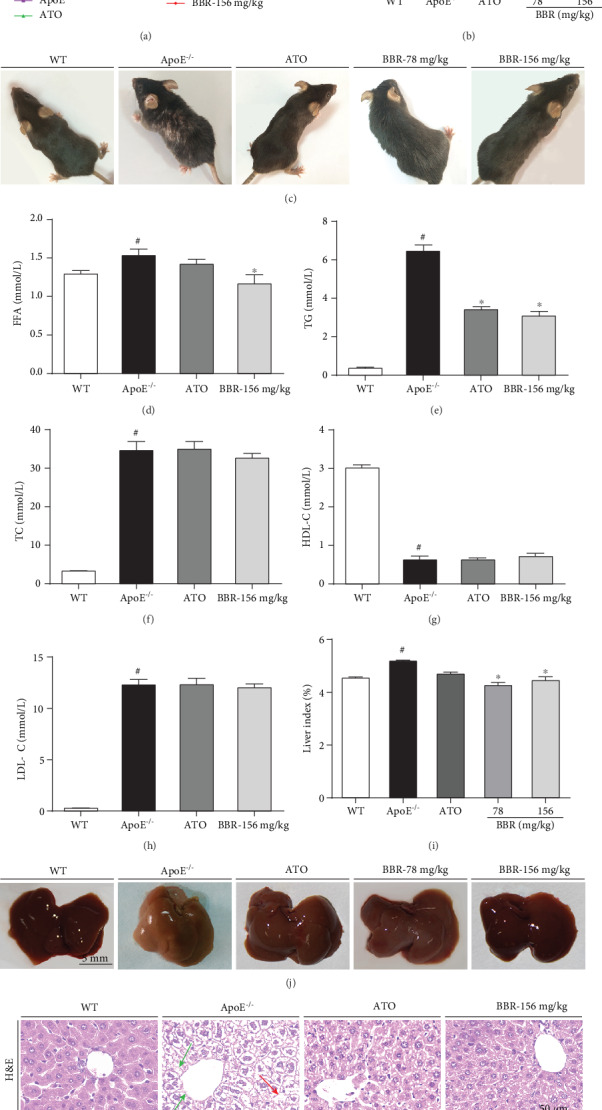
Berberine reduced serum lipid levels and antagonized hepatic lipid accumulation in ApoE^−/−^ mice. Male wild-type C57BL/6 mice fed a normal chow diet and ApoE^−/−^ mice fed a western-type diet in the presence and absence of berberine (78 and 156 mg·kg^−1^) or the presence of atorvastatin were administered by oral gavage for 12 weeks. (a) Body weight changes during the 12-week treatment (*n* = 15 in the WT, ApoE^−/−^, ATO, and BBR-156 mg·kg^−1^ groups; *n* = 7 in the BBR-78 mg·kg^−1^ group). (b) Body weight at 12 weeks of administration (*n* = 15 in the WT, ApoE^−/−^, ATO, and BBR-156 mg·kg^−1^ groups; *n* = 7 in the BBR-78 mg·kg^−1^ group). (c) Mice photos. (d–h) Serum levels of FFA, TG, TC, HDL-C, and LDL-C (*n* = 8). (i) Liver index (*n* = 12 in the WT, ApoE^−/−^, ATO, and BBR-156 mg·kg^−1^ groups; *n* = 6 in the BBR-78 mg·kg^−1^ group). (j) Liver photos. (k) Representative photomicrographs of H&E (top) and Oil Red O (bottom) staining of liver; magnification: 400x. The red arrow shows steatosis, and the green arrow shows inflammatory cell infiltrations in the liver. Data are shown as mean ± SEM. ^#^*P* < 0.05 versus WT and ^∗^*P* < 0.05 versus ApoE^−/−^. WT: wild-type; BBR: berberine; ATO: atorvastatin; FFA: free fatty acids; TG: triglyceride; TC: total cholesterol; HDL-C: high-density lipoprotein cholesterol; LDL-C: low-density lipoprotein cholesterol.

ApoE^−/−^ mice fed a western-type diet for 12 weeks were used as the atherosclerosis model, and berberine was administered by oral gavage. Throughout the course of the experiments, mouse body weights were recorded weekly (Figure 1(a)), and the weights of all the mice increased compared with that before the experiment. After 12 weeks of administration, the weight of ApoE^−/−^ mice increased significantly, and 156 mg·kg^−1^ berberine dramatically inhibited the weight increase (Figure 1(b)). With weight increase, the hair of ApoE^−/−^ mice appeared dim and depilated, which could be improved by berberine (Figure 1(c)). To confirm the hypolipidaemic effect of berberine, serum lipid profiles were determined. Compared with wild-type mice, in ApoE^−/−^ mice, the FFA, TG, TC, and LDL-C serum levels were significantly increased and the HDL-C level was significantly decreased by the western-type diet. Berberine (156 mg·kg^−1^) lowered the FFA (Figure 1(d)) and TG (Figure 1(e)) levels but had no significant effect on TC (Figure 1(f)), HDL-C (Figure 1(g)), and LDL-C (Figure 1(h)).

Compared with wild-type mice, the liver index of ApoE^−/−^ mice increased (Figure 1(i)) and the liver colour was reduced (Figure 1(j)), implying hepatic lipid accumulation. Berberine (78 and 156 mg·kg^−1^) blocked the liver colour change and reduced the liver index. In Figure 1(k), H&E staining showed slight inflammatory cell infiltration and vacuoles in hepatocytes of ApoE^−/−^ mice, indicating steatosis of the liver; Oil Red O staining showed a marked increase in fat droplets in ApoE^−/−^ mice. However, treatment with berberine or atorvastatin significantly reduced the size of lipid droplets and alleviated hepatic steatosis, suggesting that berberine antagonizes hepatic lipid accumulation.

### 3.2. Berberine Treatment Inhibited the Development of Atherosclerosis

**Figure 2:**
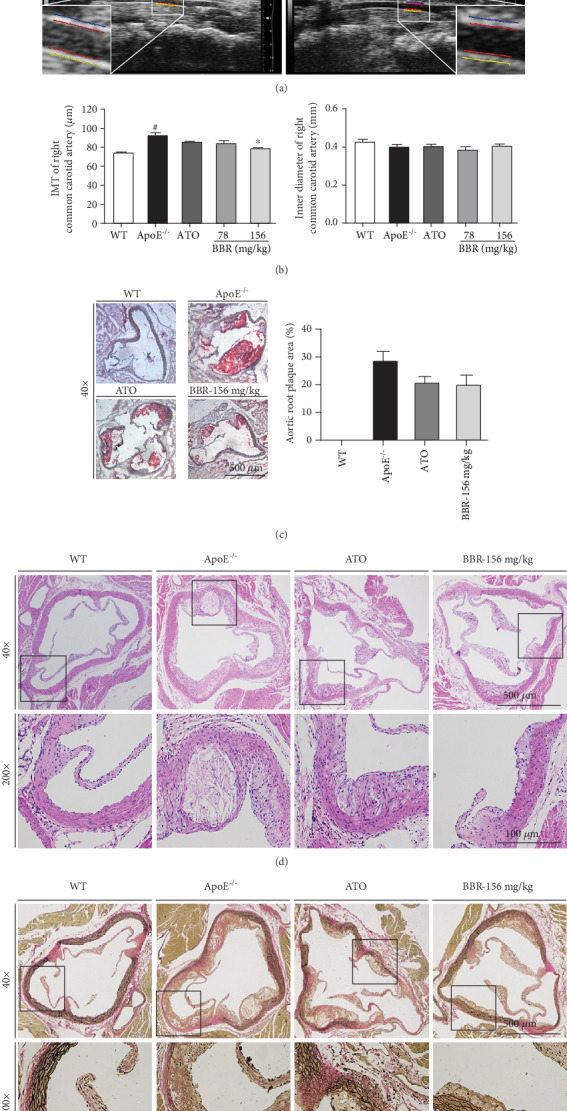
Berberine inhibited atherosclerosis in ApoE^−/−^ mice. (a) Representative ultrasound images of mice right common carotid artery. (b) IMT and inner diameter of right common carotid artery in each group of mice (*n* = 6). (c) Representative photomicrographs of Oil Red O staining in aortic root; magnification: 40x (left). Plaque areas of aortic root were expressed as percentage of total areas (right). (d) Representative photomicrographs of H&E staining in aortic root; magnification: 40x (top) and 200x (bottom). (e) Representative photomicrographs of Verhoeff-Van Gieson staining in aortic root; magnification: 40x (top) and 200x (bottom). Data are shown as mean ± SEM. ^#^*P* < 0.05 versus WT and ^∗^*P* < 0.05 versus ApoE^−/−^. WT: wild-type; BBR: berberine; ATO: atorvastatin; IMT: intima-media thickness.

At the end of the experiments, ultrasonography was used to evaluate cardiac function (Supplementary Figure 1) and carotid artery function in mice (Figure 2(a)). The intima-media thickness (IMT) of the carotid artery in ApoE^−/−^ mice increased significantly, and 156 mg·kg^−1^ berberine treatment for 12 weeks improved the IMT thickening, but there was no difference in the carotid artery inner diameter in mice of each group (Figure 2(b)). To further determine the antiatherosclerotic effect of berberine, we performed Oil Red O, H&E, and VVG staining of the aortic root. As shown in Figure 2(c) (Oil Red O staining), apparent lipid accumulation and plaque formation were present in the aortic root of ApoE^−/−^ mice fed the western-type diet, whereas no lipid plaque accumulation was found in wild-type mice. Administration of berberine or atorvastatin showed a tendency to reduce the plaque area. As shown in Figure 2(d) (H&E staining), compared with wild-type mice, aortic intimal hyperplasia, lipid deposition, and plaque formation were observed in the ApoE^−/−^ mice, accompanied by inflammatory cell infiltration. The sinus lesions were improved in berberine-treated ApoE^−/−^ mice, demonstrating the antiatherosclerotic effect of berberine.  Figure 2(e) (VVG staining) shows arterial lesions in ApoE^−/−^ mice, displaying an apparent disruption, decrease, and even loss of elastin fibres. However, berberine prevented these pathologies and increased the elastin content in the arterial wall.

### 3.3. Berberine Improved Endothelial Dysfunction in ApoE^−/−^ Mice

**Figure 3:**
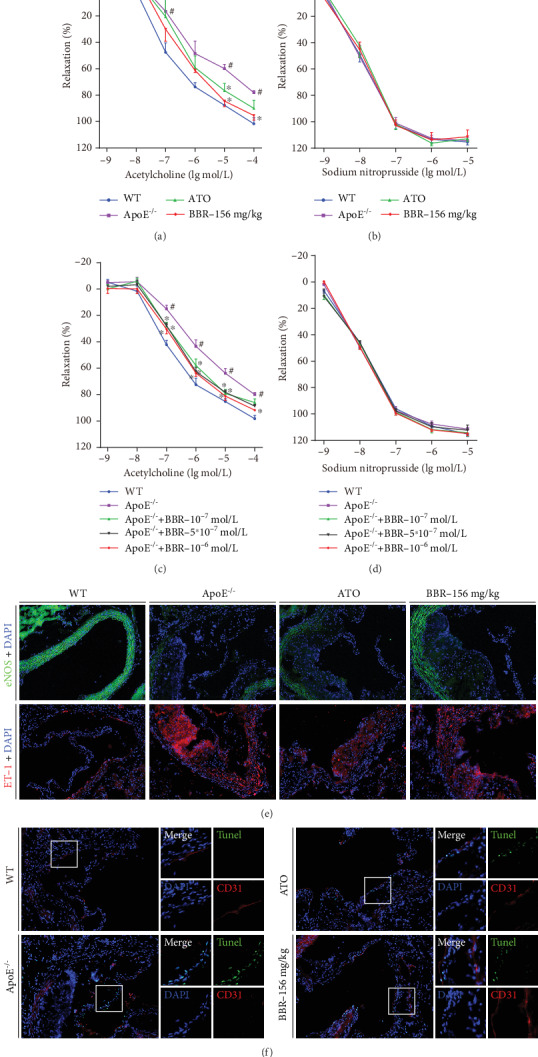
Berberine improved endothelial dysfunction in vivo and in vitro. (a and b) ApoE^−/−^ mice fed with western-type diet were intragastrically gavaged with berberine (156 mg·kg^−1^) or atorvastatin for 12 weeks. After the end of the experiment, the mice were sacrificed and the thoracic aorta was isolated for aortic ring assay. (a) Endothelium-dependent relaxation induced by acetylcholine. (b) Endothelium-independent relaxation induced by sodium nitroprusside. (c and d) The isolated mice aortic rings were preincubated with berberine (10^−7^, 5 × 10^−7^, 10^−6^ M) for 30 min. The endothelium-dependent relaxation induced by acetylcholine (c) and endothelium-independent relaxation induced by sodium nitroprusside (d) were assayed by organ chamber. (e) Expression of eNOS (top, green) and ET-1 (bottom, red) in mice aortic root was determined by immunofluorescent staining. Nuclei were stained with DAPI (blue). Magnification: 200x. (f) Representative images of double immunofluorescence staining for CD31 (red) and TUNEL (green) in mice aortic root. Nuclei were stained with DAPI (blue). Magnification: 200x. Data are shown as mean ± SEM, *n* = 5‐6. ^#^*P* < 0.05 versus WT and ^∗^*P* < 0.05 versus ApoE^−/−^. WT: wild-type; BBR: berberine; ATO: atorvastatin; eNOS: endothelial nitric oxide synthase; ET-1: Endothelin 1; DAPI: 4′,6-diamidino-2-phenylindole.

Thoracic aortic rings were isolated to investigate whether berberine can protect against endothelial dysfunction in ApoE^−/−^ mice fed a high-fat diet. Endothelial function was determined by measuring endothelium-dependent vasodilatation in response to ACh. As shown in Figure 3(a), after 12 weeks of administration, endothelium-dependent vasodilatation induced by ACh was significantly impaired in ApoE^−/−^ mice compared with wild-type mice. Importantly, the impairment of ACh-induced vasodilatation was restored by administration of berberine. However, there was no difference in SNP-induced endothelial-independent vasodilatation between the groups (Figure 3(b)), demonstrating that the protective effect of berberine on the aorta involves maintaining endothelial function. Similarly, in vitro experiments showed that aortic endothelium-dependent relaxation was strikingly preserved by berberine in ApoE^−/−^ mice (Figure 3(c)), and endothelium-independent vasodilatation was not altered in any of the groups (Figure 3(d)), indicating a significant improvement in endothelial function by berberine.

To further evaluate vascular endothelial function, aortic root eNOS, ET-1 immunofluorescence, and TUNEL staining were performed. Representative images are shown in Figures 3(e) and 3(f). Downregulated expression of eNOS (green) and overexpression of ET-1 (red) in the aortic root were observed in ApoE^−/−^ mice, and berberine treatment reversed the expression of eNOS and ET-1 in part (Figure 3(e)).  Figure 3(f) shows increased apoptotic cells (green) in the sinus lesion area in ApoE^−/−^ mice compared with the wild-type mice, but berberine administration reduced the number of TUNEL-positive cells. Together, these data indicate the protective effect of berberine on the aorta of ApoE^−/−^ mice.

### 3.4. Berberine Attenuated Aortic Oxidative Stress in ApoE^−/−^ Mice

**Figure 4:**
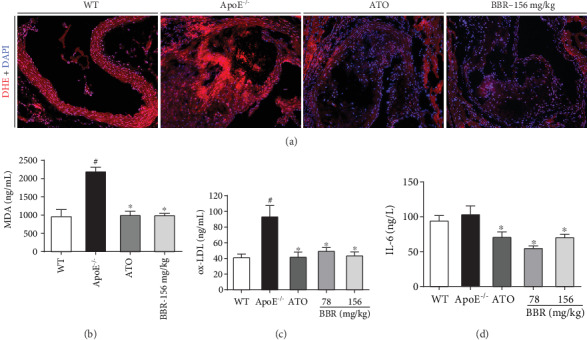
Berberine attenuated aortic oxidative stress in ApoE^−/−^ mice. (a) Representative images of dihydroethidium staining (red) in mice aortic root. Nuclei were stained with DAPI (blue). Magnification: 200x. (b) Serum levels of MDA (*n* = 6). (c) Serum levels of ox-LDL (*n* = 5). (d) Serum levels of IL-6 (*n* = 5). Data are shown as mean ± SEM. ^#^*P* < 0.05 versus WT and ^∗^*P* < 0.05 versus ApoE^−/−^. WT: wild-type; BBR: berberine; ATO: atorvastatin; DHE: dihydroethidium; MDA: malondialdehyde; ox-LDL: oxidized low-density lipoprotein; IL-6: interleukin-6.

DHE staining was performed to detect endothelial ROS generation to evaluate the effect of berberine on oxidative stress. Compared with wild-type mice, DHE fluorescence intensity increased in ApoE^−/−^ mice, indicating that the total aortic ROS formation increased, whereas berberine treatment reduced endothelial fluorescence (Figure 4(a)). Serum levels of the oxidative stress products MDA and ox-LDL were remarkably higher in ApoE^−/−^ mice than in wild-type mice. After treatment with berberine or atorvastatin, the alterations in MDA and ox-LDL levels were significantly inhibited (Figures 4(b) and 4(c)). Furthermore, administration of berberine decreased the level of the inflammatory cytokine IL-6 in ApoE^−/−^ mice (Figure 4(d)).

### 3.5. TMT Protein Profiling

**Figure 5:**
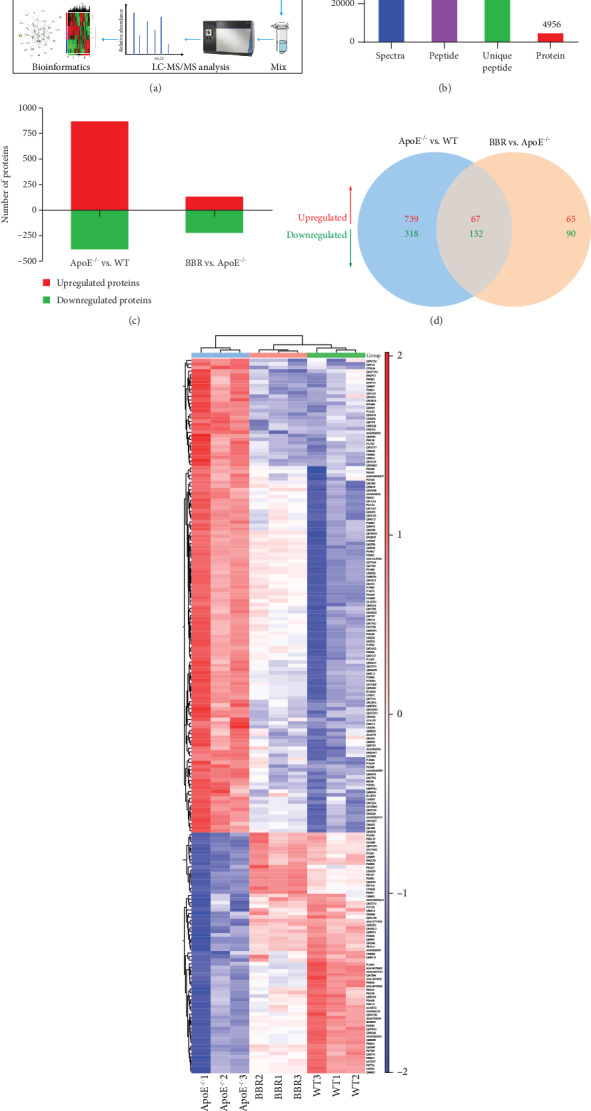
TMT-based quantification of the aortic proteomes. (a) Workflow for TMT protein profiling of aorta in this study. (b) Protein identification results. 4,956 proteins (unique peptides ≥ 1) were identified from 28,957 unique peptides. (c) The number of up- and downregulated proteins in each comparison group. (d) Venn diagram of the distribution and overlaps of differentially expressed proteins among the two comparison groups. (e) Hierarchical clustering of 199 common differentially expressed proteins. WT: wild-type; BBR: berberine.

To obtain a global overview of proteins regulated by berberine and better understand the mechanism by which berberine protects the aorta of ApoE^−/−^ mice, a proteomic profiling analysis based on the TMT technique was carried out. The workflow of this study is shown in Figure 5(a). Through LC-MS/MS analysis, a total of 59,751 spectra were obtained, corresponding to 31,414 peptides. In total, 4,956 proteins (unique peptides ≥ 1) were identified from 28,957 unique peptides (Figure 5(b)), and 4,841 proteins were successfully quantified (Supplementary Table 1). The proteins that exhibited a *P* value < 0.05 and a ratio − fold change > 1.2 or <0.83 were defined as differentially expressed proteins. Compared with wild-type mice, 871 upregulated proteins and 385 downregulated proteins were identified in ApoE^−/−^ mice fed the western-type diet, whereas berberine treatment upregulated 132 proteins and downregulated 222 proteins (Figure 5(c)). In Figure 5(d), the Venn diagram shows the distribution and overlap of differentially expressed proteins among the two comparison groups. There were 199 common differentially expressed proteins among these comparison groups, including 67 upregulated proteins and 132 downregulated proteins. The expression of these common differentially expressed proteins was clustered through hierarchical clustering (Figure 5(e)), and the top 20 are shown in Table 1.

### 3.6. Bioinformatics Analysis of Differentially Expressed Proteins and Western Blotting Validation

**Figure 6:**
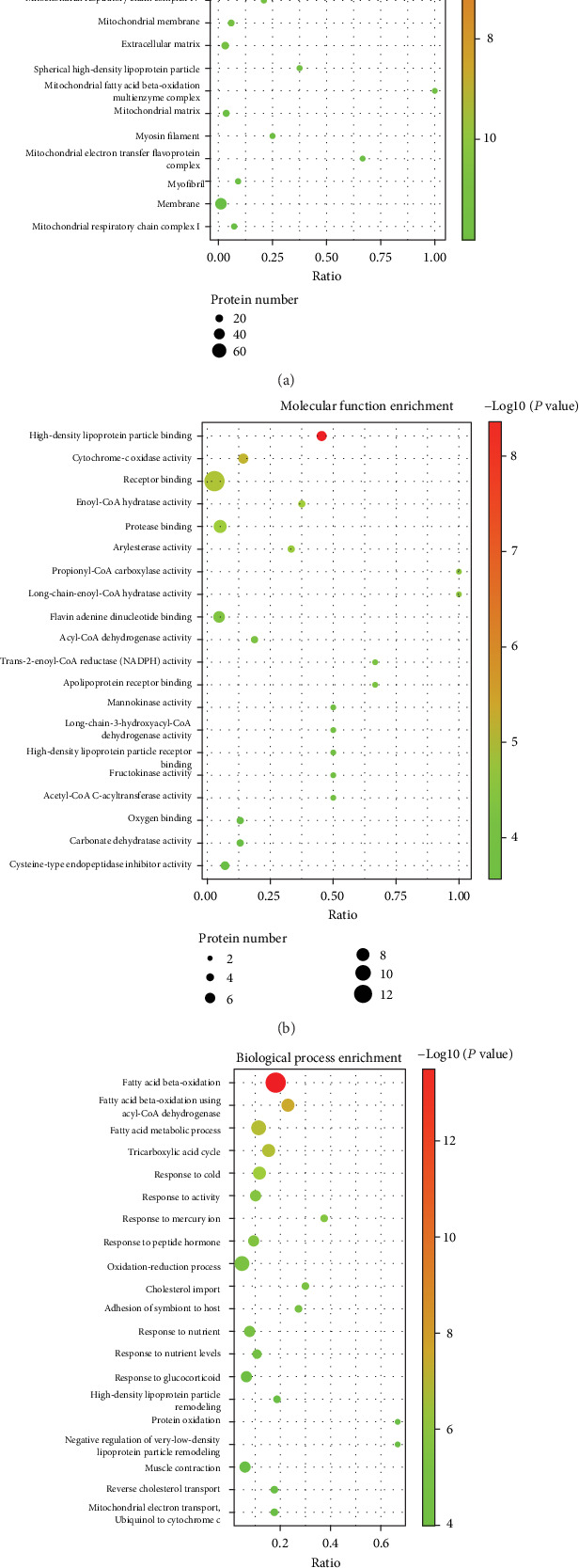
Gene ontology (GO) enrichment analysis of 199 common differentially expressed proteins. (a) Top 20 cellular component enrichment terms. (b) Top 20 molecular function enrichment terms. (c) Top 20 biological process enrichment terms. The size of the spot represents the number of genes corresponding to each term, and the colour represents −log(*P* value).

To clarify the biological significance of the 199 differentially expressed proteins, we first performed GO enrichment analysis to annotate the functions of these proteins according to cellular component, molecular function and biological process. 628 GO terms with *P* < 0.05 were significantly enriched, 79 in cellular component, 160 in molecular function, and 389 in biological process. The data of GO analysis are shown in Supplementary Table 2 and the top 20 terms of each category are shown in Figure 6. From the cellular component enrichment terms, it can be noted that the differentially expressed proteins are likely primarily located in mitochondrion (Figure 6(a)). In molecular function enrichment, high-density lipoprotein particle binding was the top enriched term, while receptor binding contained the most differentially expressed proteins (Figure 6(b)). Regarding biological process, the differentially expressed proteins were remarkably enriched in fatty acid beta-oxidation, which might play important roles in berberine protection of the aorta in atherosclerosis (Figure 6(c)).

**Figure 7:**
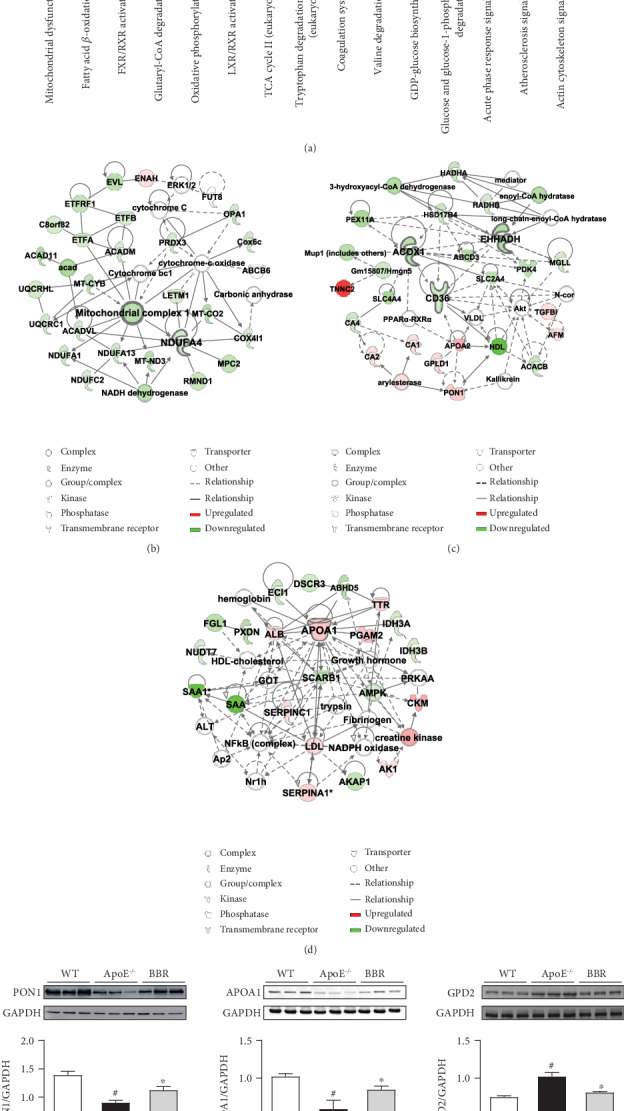
Ingenuity Pathway Analysis (IPA) and western blotting validation of common differentially expressed proteins. (a) Top 15 canonical pathways according to IPA. The blue bars represent −log(*P* value), and the orange points denote the ratio of differentially expressed proteins. (b–d) Top 3 networks generated by IPA. Network 1 is involved in metabolic disease, developmental disorder, and hereditary disorder (b); Network 2 is involved in energy production, lipid metabolism, and small molecule biochemistry (c); Network 3 is involved in lipid metabolism, small molecule biochemistry, and vitamin and mineral metabolism (d). (e) Validation of differentially expressed proteins PON1, GPD2, and APOA1 by western blotting. Data are shown as mean ± SEM, *n* = 5‐6. ^#^*P* < 0.05 versus WT and ^∗^*P* < 0.05 versus ApoE^−/−^. WT: wild-type; BBR: berberine; PON1: paraoxonase 1; APOA1: apolipoprotein A I; GPD2: glycerol-3-phospate dehydrogenase 2.

Next, IPA was performed for further biological function investigation.  Figure 7(a) shows the top 15 canonical pathways according to *P* value. Among them, mitochondrial dysfunction was the most significant pathway and was related to oxidative stress and metabolic disease. The fatty acid *β*-oxidation I (top 2, −log(*P* value) = 8.14), FXR/RXR activation (top 3, −log(*P* value) = 6.96), and glutaryl-CoA degradation (top 4, −log(*P* value) = 6.8) pathways were all involved in regulation of lipid metabolism. The entire detailed canonical pathway list is provided in Supplementary Table 3. Additionally, network analysis identified complex interactions (either direct or indirect) between these proteins. The top 3 networks of all 15 networks (Supplementary Table 4) according to score are shown in Figures 7(b)–7(d). Network 1 (Figure 7(b)), in which NDUFA4 (NDUFA4 mitochondrial complex associated) and Mitochondrial complex 1 are the core proteins, is primarily involved in metabolic disease, developmental disorder, and hereditary disorder. Network 2 (Figure 7(c)) mediates biological processes, such as energy production, lipid metabolism, and small molecule biochemistry, and ACOX1 (acyl-CoA oxidase 1), CD36, and EHHADH (enoyl-CoA hydratase and 3-hydroxyacyl CoA dehydrogenase) are the core proteins. In Network 3 (Figure 7(d)), APOA1 may play a key role. This network is primarily related to lipid metabolism, small molecule biochemistry, and vitamin and mineral metabolism. IPA was also adopted for grouping of differentially expressed proteins into three categories: “Diseases and Disorders,” “Molecular and Cell Functions,” and “Physiological System Development and Function” (Supplementary Figure 2). In “Diseases and Disorders,” metabolic disease was found to be the top ranked, and cardiovascular disease was one of the related diseases (Supplementary Figure 2a). The molecular profiles of the top 4 categories in metabolic disease and cardiovascular disease are shown in Supplementary Figure 3.

Three proteins, including PON1, APOA1, and GPD2, involved in atherosclerosis, cardiovascular disease, and lipid metabolism, were selected for validation via western blotting. The expression levels of PON1 and APOA1 were found to be remarkably downregulated in ApoE^−/−^ mice compared with wild-type mice, whereas GPD2 showed significant upregulation. Berberine treatment upregulated the expression of PON1 and APOA1 and downregulated the expression of GPD2 (Figure 7(e)). These results were consistent with the results observed in the TMT-based proteomics analysis.

## 4. Discussion

Hyperlipidaemia, characterized by dyslipidaemia, including elevated blood levels of TG, TC, and LDL-C and a decreased HDL-C level, is a major risk factor for atherosclerosis [25]. Regulation of disorder of lipid metabolism is one method used to control atherosclerosis development. Our results showed that berberine treatment significantly lowered the TG and FFA levels in western-type diet-induced ApoE^−/−^ mice, indicating the lipid-lowering effect of berberine. In addition, the liver is a crucial organ of lipid metabolism and is responsible for maintaining the homeostasis of cholesterol and LDL [26]. We observed hepatic lipid accumulation. As shown in Figures 1(i)–1(k), berberine alleviated hepatic steatosis and reduced liver lipid accumulation, which indicates that berberine may have a potential therapeutic effect on fatty liver and further investigation is needed. Taken together, these data suggest that the protective effect on the aorta and the antiatherosclerotic effect of berberine might be partially due to its regulation of lipid metabolism.

Endothelial dysfunction is an early step in atherosclerosis [6] and an independent predictor of future cardiovascular events [27]. Thus, targeting endothelial dysfunction represents an attractive pharmacological approach [28]. Impaired endothelium-dependent vasodilatation is the hallmark of endothelial dysfunction [29]. Improving endothelium-dependent vasodilatation and restoring endothelial function in patients with atherosclerosis may prevent cardiovascular events [30]. In addition, preventing impairment of endothelium-dependent relaxation was found to attenuate atherosclerosis in ApoE^−/−^ mice [31]. Our results revealed impaired endothelium-dependent vasodilatation induced by ACh in ApoE^−/−^ mice fed a western-type diet, suggesting endothelial dysfunction in ApoE^−/−^ mice. However, berberine intervention significantly restored endothelium-dependent vasodilatation in vivo and in vitro, indicating the protective effect of berberine on endothelial function. Many factors, including dyslipidaemia, oxidative stress, and inflammation, are involved in the progression of endothelial dysfunction. Increased FFA concentration in plasma results in an increase in ROS generation [32], while elevated circulating LDL deposits in the subendothelial matrix and is oxidized to form ox-LDL through increased ROS production. Ox-LDL injures endothelial cells directly and contributes to endothelial dysfunction via overexpression of lectin-like oxidized low-density lipoprotein receptor-1 (LOX-1), which induces a further rise in intracellular ROS [33–35]. ROS oxidize tetrahydrobiopterin, leading to eNOS uncoupling [36], and react with nitric oxide (NO) to form peroxynitrite (ONOO^−^), thus reducing NO generation and bioavailability and accelerating endothelial dysfunction [37]. On the other hand, ROS are able to trigger activation of the transcription factor nuclear factor kappa B (NF-*κ*B) [38], which can activate a variety of proinflammatory cytokines, such as IL-6, IL-8, E-selectin, ICAM-1, and VCAM-1 [39, 40], thereby activating inflammation. In turn, IL-6 induces oxidative stress and endothelial dysfunction through overexpression of angiotensin II type 1 receptor in the atherosclerosis process [41]. A vicious circle exists between oxidative stress and inflammation in atherosclerosis [42]. In the current study, decreased ROS generation in the aortic root and lower serum levels of MDA, ox-LDL, and IL-6 were detected in the berberine-treated ApoE^−/−^ mice, demonstrating the anti-inflammatory and antioxidative properties of berberine, which may be the mechanism by which berberine improves endothelial dysfunction.

Carotid IMT is a surrogate marker for atherosclerosis [43]. An increase in carotid IMT is related to endothelial dysfunction, and both of them, respectively, assess the atherosclerotic process from different aspects of anatomy and function [44]. Ultrasonography is a noninvasive approach based on high-resolution scanning and a computerized image-analysis system and has been a powerful tool to assess vascular atherosclerosis in mice. In the present study, we used long-axis views obtained with an ultrasound imaging system to measure carotid IMT. The ultrasound results showed that berberine significantly reduced IMT in the carotid artery, indicating that berberine could improve arterial wall lesions. We further investigated the pathological changes in the aortic root in ApoE^−/−^ mice, which exhibited an obvious accumulation of lipid, plaque formation, and severe atherosclerotic lesions, suggesting successful establishment of the atherosclerosis model. Treatment with berberine alleviated the severity of the sinus atherosclerotic lesions in ApoE^−/−^ mice. These results demonstrate that berberine treatment inhibits atherosclerosis development.

To further reveal the mechanism of berberine in ameliorating atherosclerosis and protecting the aorta of ApoE^−/−^ mice, a TMT-based quantitative proteomics analysis was performed to globally screen berberine-regulated proteins, followed by bioinformatics analysis and validation. Based on the data from TMT protein profiling and GO analysis, we found 199 proteins regulated by berberine in the treatment of atherosclerosis, which are likely primarily located in mitochondrion and participate in the biological process of fatty acid beta-oxidation (Figure 6), indicating that berberine might protect the aorta by regulating mitochondrial function and lipid metabolism. IPA canonical pathways analysis showed that mitochondrial dysfunction (−log(*P* value) = 8.66) was the most significant pathway related to the differentially expressed proteins (Figure 7(a)). Mitochondria contain a multitude of redox carriers and are the main sites of aerobic respiration, producing adenosine triphosphate (ATP) via oxidative phosphorylation; thus, mitochondria are known as the “power house” of cells. In addition to producing energy, mitochondria are involved in regulating many other cellular processes, such as calcium signalling [45], cell death [46], and inflammation [47]. Moreover, mitochondria are an important source of ROS, approximately 90% of which can be traced back to mitochondria [48]. Under the stimulation of risk factors, such as hypercholesterolemia, hyperlipidaemia, and hypoxia, the mitochondrial membrane potential can be altered, triggering excessive ROS production in the mitochondria, which results in mitochondrial damage and dysfunction [49]. In turn, mitochondrial dysfunction leads to more ROS production, and the enhanced ROS production induces endothelial dysfunction by uncoupling eNOS and triggering inflammation. Mitochondrial dysfunction is present in human and mouse atherosclerosis and promotes the development of atherosclerosis by affecting endothelial function, vascular smooth muscle cell proliferation or apoptosis, and macrophage polarization [50, 51]. As a consequence, mitochondria-targeted intervention may provide a novel therapeutic strategy for atherosclerosis. It has been found that chlorogenic acid can mitigate ox-LDL-induced endothelial oxidative stress and mitochondrial dysfunction by modulating the SIRT1/AMPK/PGC-1 pathway, thus providing beneficial effects in atherosclerosis [52]. Our results showed that the differentially expressed proteins regulated by berberine were most related to mitochondrial dysfunction, suggesting that regulation of mitochondrial dysfunction may be one of the mechanisms by which berberine protects the atherosclerotic aorta.

The IPA network analysis showed that APOA1 accounted for a large proportion of Network 3 and was upregulated by berberine (Figure 7(d)). APOA1 is the major protein component of HDL, which determines the levels of HDL in the plasma [53]. Elevated APOA1 levels are strongly associated with a reduced risk of cardiovascular events in patients [54], and a damaging mutation in APOA1 confers an increased risk of atherosclerosis [55]. Animal studies have also shown that APOA1 inhibits the progression of atherosclerosis. Somatic gene transfer of human APOA1 reduced atherosclerosis progression in human APOA1-transgenic mice and ApoE^−/−^ mice [56]. Infusions of synthetic HDL containing trimeric human APOA1 stabilized atherosclerotic plaques in hypercholesterolemic rabbits [57]. Guo et al. found that perhexiline reduced atherosclerosis by regulating APOA1 transcription and increasing the APOA1 level in mice [58]. Consequently, increasing APOA1 synthesis may become a new approach for treating atherosclerosis. Herein, APOA1 expression was downregulated in ApoE^−/−^ mice, and the expression downregulation was reversed by berberine. The “Diseases and Disorders” analysis in the IPA showed that APOA1 was associated with disorder of lipid metabolism, coronary disease, acute coronary syndrome, and occlusion of blood vessels (Supplementary Figure 3). These results indicate that berberine has a regulatory effect on APOA1, which might be a potential target of berberine.

## 5. Conclusions

**Figure 8:**
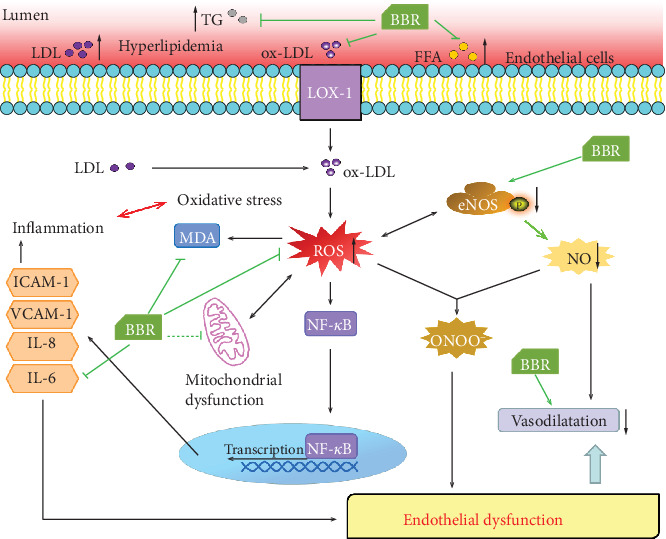
Schematic diagram of the mechanisms by which berberine ameliorates endothelial dysfunction and protects against atherosclerosis. In atherosclerotic mice, berberine regulated lipid metabolism, alleviated atherosclerotic lesions, attenuated oxidative stress and inflammation, and ameliorated endothelial dysfunction, thus restoring endothelium-dependent vasodilatation to protect against atherosclerosis. Regulation of mitochondrial dysfunction might be potential mechanisms underlying this protection.

In summary (Figure 8), our study demonstrated that berberine can ameliorate endothelial dysfunction to protect against atherosclerosis by restoring endothelium-dependent vasodilatation, attenuating oxidative stress and inflammation, regulating lipid metabolism, and alleviating atherosclerotic lesions. Regulation of mitochondrial dysfunction and targeting of APOA1 might be potential mechanisms underlying this protection. The exact mechanisms remain to be further studied and characterized, but together, the findings presented here provide novel insight into the molecular antiatherosclerosis mechanism of berberine and evidence to support the potential of berberine as a promising therapeutic candidate for atherosclerosis.

## Figures and Tables

**Table 1:** List of top 20 common differentially expressed proteins.

Accession	Gene name	Description	Log ratio	*P* value
E9QNH7	Acbd5	Acyl-CoA-binding domain-containing protein 5	-0.27	1.56*E*‐05
Q5SX39	Myh4	Myosin-4	1.53	4.77*E*‐05
P20801	Tnnc2	Troponin C, skeletal muscle	2.17	5.20*E*‐05
P97457	Mylpf	Myosin regulatory light chain 2, skeletal muscle isoform	2.19	5.69*E*‐05
Q5SX40	Myh1	Myosin-1	1.74	6.95*E*‐05
P05977	Myl1	Myosin light chain 1/3, skeletal muscle isoform	1.76	6.96*E*‐05
P07310	Ckm	Creatine kinase M-type	0.87	0.0001716
G3UW82	Myh2	MCG140437, isoform CRA_d	1.28	0.0003
Q8VCU2	Gpld1	Glycosylphosphatidylinositol-specific phospholipase D1	0.38	0.000313
A0A1Y7VJN6	Ighg3	Immunoglobulin heavy constant gamma 3	0.60	0.000317
Q8K2C8	Gpat4	Glycerol-3-phosphate acyltransferase 4	-0.63	0.000346
Q8CI78	Rmnd1	Required for meiotic nuclear division protein 1 homolog	-0.38	0.000385
O70250	Pgam2	Phosphoglycerate mutase 2	0.60	0.000433
A0A0R4J0E1	Fgl1	Fibrinogen-like protein 1	-0.48	0.000537
P32261	Serpinc1	Antithrombin-III	0.36	0.000669
E9QLZ9	Enah	Protein-enabled homolog	0.28	0.000699
Q9Z2C8	Ybx2	Y-box-binding protein 2	-0.78	0.000764
Q9WTS2	Fut8	Alpha-(1,6)-fucosyltransferase	-0.43	0.000778
Q9Z211	Pex11a	Peroxisomal membrane protein 11A	-0.61	0.000886
G3X9F4	Tmem143	Transmembrane protein 143	-0.34	0.0012204

## Data Availability

The data used to support the findings of this study are included within the article.

## Abbreviations

ACh: Acetylcholine
APOA1: Apolipoprotein A I
ApoE^−/−^: Apolipoprotein E-deficient
ATO: Atorvastatin
BBR: Berberine
DAPI: 4′,6-Diamidino-2-phenylindole
DHE: Dihydroethidium
eNOS: Endothelial nitric oxide synthase
ET-1: Endothelin 1
FFA: Free fatty acids
GAPDH: Glyceraldehyde-3-phosphate dehydrogenase
GO: Gene Ontology
GPD2: Glycerol-3-phospate dehydrogenase 2
HDL-C: High-density lipoprotein cholesterol
H&E: Haematoxylin-eosin
ICAM-1: Intercellular adhesion molecule-1
IL-6: Interleukin-6
IMT: Intima-media thickness
IPA: Ingenuity pathway analysis
K-H: Krebs-Henseleit
LC-MS/MS: Liquid chromatography-tandem mass spectrometry
LDL-C: Low-density lipoprotein cholesterol
MDA: Malondialdehyde
MS: Mass spectrometry
NE: Norepinephrine
NO: Nitric oxide
Ox-LDL: Oxidized low-density lipoprotein
PON1: Paraoxonase 1
ROS: Reactive oxygen species
SNP: Sodium nitroprusside
TC: Total cholesterol
TG: Triglyceride
TMT: Tandem mass tags
TNF-*α*: Tumor necrosis factor *α*
TUNEL: Terminal deoxynucleotidyl transferase-mediated dUTP nick-end labelling
VCAM-1: Vascular cell adhesion molecule-1
VVG: Verhoeff-Van Gieson
WT: Wild type.

## Supplementary Materials

Supplementary 1 Supplementary Table 1: the list of quantitative proteins.

Supplementary 2 Supplementary Table 2: the data of GO enrichment analysis including cellular component, molecular function, and biological process.

Supplementary 3 Supplementary Table 3: the list of canonical pathways by IPA.

Supplementary 4 Supplementary Table 4: the list of networks by IPA.

Supplementary 5 Supplementary Figure 1: effects of BBR on cardiac function. Male wild-type (WT) C57BL/6 mice were fed with normal chow diet, and ApoE^−/−^ mice were fed with western-type diet in the presence and absence of berberine (BBR, 78 and 156 mg·kg^−1^) or the presence of atorvastatin (ATO). After 12 weeks of administration, echocardiography was performed. (a) Representative echocardiograms (M-mode) showing the wall motion. (b) Peak Vel echocardiograms on Doppler of the left ventricular outflow tract. (c) Echocardiographic measurements of ejection fraction (EF), fractional shortening (FS), peak Vel and peak pressure of left ventricular outflow tract in different groups. Data are shown as mean ± SEM. ^#^*P* < 0.05 versus WT, ^∗^*P* < 0.05 versus ApoE^−/−^. Supplementary Figure 2: disease and function analysis of common differentially expressed proteins according to IPA. The differentially expressed proteins were grouped into three categories: diseases and disorders (a), molecular and cell functions (b), and physiological system development and function (c). Supplementary Figure 3: the molecular profiles of top 4 categories in metabolic disease and cardiovascular disease identified by IPA. (a) The molecular profiles of disorder lipid metabolism, fatty acid oxidation disorder, enzymopathy, and hepatic steatosis in metabolic disease. (b) The molecular profiles of coronary disease, abnormal morphology of the heart, acute coronary syndrome, and occlusion of blood vessel in cardiovascular disease.
