# Supplementary material for: Improvement of Endothelial Dysfunction of Berberine in Atherosclerotic Mice and Mechanism Exploring through TMT-Based Proteomics
Source: Oxid Med Cell Longev. 2020 May 31;2020:8683404. doi: 10.1155/2020/8683404 (PMC7284929; doi:10.1155/2020/8683404)
Supplement: Supplementary 4 — Supplementary Table 4: the list of networks by IPA. [file 8683404.f4.docx]

**Supplementary Table 4. The list of networks by IPA**

| ID | Score | Focus Molecules | Top Diseases and Functions | Molecules in Network |
| --- | --- | --- | --- | --- |
| 1 | 53 | 27 | Metabolic Disease, Developmental Disorder, Hereditary Disorder | **ABCB6**, acad, **ACAD11**, **ACADM**, **ACADVL**, **C8orf82**, Carbonic anhydrase, **COX4I1**, Cytochrome bc1, cytochrome C, cytochrome-c oxidase, **ENAH**, ERK1/2, **ETFA**, **ETFB**, **ETFRF1**, **EVL**, **FUT8**, **LETM1**, Mitochondrial complex 1, **MPC2**, **MT-CO2**, **MT-CYB**, **MT-ND3**, **Cox6c**, NADH dehydrogenase, **NDUFA1**, **NDUFA13**, **NDUFA4**, **NDUFC2**, **OPA1**, **PRDX3**, **RMND1**, **UQCRC1**, **UQCRHL** |
| 2 | 45 | 24 | Energy Production, Lipid Metabolism, Small Molecule Biochemistry | 3-hydroxyacyl-CoA dehydrogenase, HDL, **ABCD3**, **ACACB**, **ACOX1**, **AFM**, Akt, **APOA2**, arylesterase, **CA1**, **CA2**, **CA4**, **CD36**, **EHHADH**, enoyl-CoA hydratase, **Gm15807/Hmgn5**, **GPLD1**, **HADHA**, **HADHB**, VLDL, **HSD17B4**, Kallikrein, long-chain-enoyl-CoA hydratase, mediator, **MGLL**, **Mup1 (includes others)**, N-cor, **PDK4**, **PEX11A**, **PON1**, PPARα-RXRα, **SLC2A4**, **SLC4A4**, **TGFBI**, **TNNC2** |
| 3 | 33 | 19 | Lipid Metabolism, Small Molecule Biochemistry, Vitamin and Mineral Metabolism | **ABHD5**, **AK1**, **AKAP1**, **ALB**, ALT, AMPK, Ap2, **APOA1**, **CKM**, creatine kinase, **DSCR3**, **ECI1**, **FGL1**, Fibrinogen, GOT, Growth hormone, HDL-cholesterol, hemoglobin, **IDH3A**, **IDH3B**, LDL, NADPH oxidase, NFkB, Nr1h, **NUDT7**, **PGAM2**, PRKAA, **PXDN**, SAA, **SAA1**, **SCARB1**, **SERPINA1**, **SERPINC1**, trypsin, **TTR** |
| 4 | 30 | 18 | Cancer, Dermatological Diseases and Conditions, Organismal Injury and Abnormalities | 26s Proteasome, **ACSL5**, Actin, ATPase, Calcineurin protein(s), CaMKII, **CPT1B**, **EHD4**, ERK, F Actin, **FAS**, Filamin, HLA-DR, **HLA-DRB5**, **MB**, MEF2, MHC Ⅱ, Mlc, **MYH1**, **MYH2**, **MYH4**, **MYH6**, **MYL1**, **MYLPF**, Myosin, **NEFL**, **Nefm**, Nfat (family), **PCCA**, **PCCB**, **PVALB**, Rock, **SFXN5**, TCF, Tnf (family) |
| 5 | 26 | 16 | Cardiac Damage, Cardiovascular Disease, Organismal Injury and Abnormalities | Alpha Actinin, calpain, collagen, Collagen type Ⅰ, Collagen type Ⅲ, Collagen type Ⅳ, Collagen(s), **CSRP2**, **DLAT**, **DLST**, elastase, Fibrin, **GP1BB**, **GPD2**, **HIBADH**, Ige, Integrin, JINK1/2, Jnk, **KNG1**, Mmp, **NF2**, **OGDH**, PDGF BB, PLC gamma, **VWF**, **POSTN**, **RBBP9**, **SERPINA6**, **SERPINE1**, **SLC25A22**, Smad2/3, STAT5a/b, Tgf beta, **TRAP1**, |
| 6 | 26 | 16 | Gastrointestinal Disease, Organismal Injury and Abnormalities, Cellular Assembly and Organization | ADRB, **AGPS**, **ARPC4**, **BSG**, caspase, Cg, **DPP4**, **ECH1**, EGLN, **FETUB**, **FLNA**, Focal adhesion kinase, FSH, **GBP6**, **GSTA3**, Hsp90, Lh, **LSS**, Mapk, Mek, MTORC1, **NUDT13**, **PDLIM5**, **PDLIM7**, Pkc(s), PLC, Pld, **S100A9**, Serine Protease, **SORBS2**, Sos, TCR, **TH**, TSH, Vegf |
| 7 | 26 | 16 | Skeletal and Muscular Disorders, Nervous System Development and Function, Organ Development | 20-hydroxyeicosatetraenoic acid, ADPGK, AGT, **ALDH3A1**, ATG101, **C6orf136**, **Cox7c**, DMD, **FAM213A**, FANCD2, FAR1, **HNRNPAO**, **Ighg3**, IGKC, **Igkv5-48**, LDB3, LDHC, LMAN2, **LONP2**, Loxhdl, **LRRN4**, **MYH1**, **MYOM3**, **PGM5**, PLK1, **PYGM**, RAD51B, RPA1, SGCG, SMCR8, **Sp100**, SPIN1, **SUCLA2**, Uba52, **YBX2** |
| 8 | 24 | 15 | Cell Cycle, Gene Expression, Connective Tissue Development and Function | **ACBD5**, **ADHFE1**, **VPS13D**, BAZ1B, **Ces1b/Ces1c**, COPS5, Cox5b, **CRIP1**, **DDO**, EED, ESR1, FZD2, **GFM2**, GHR, GLMN, GNAQ, HENMT1, IGF1R, **Igkv12-44**, **Igkv4-55**, IL4, INPP1, KCTD6, **MRPL15**, NR4A2, **PECR**, PON3, **SLC25A20**, SMARCA4, SMPD3, **TCEAL5**, **TMEM135**, **TMEM143**, UROS, VOPP1 |
| 9 | 22 | 14 | Gastrointestinal Disease, Hepatic System Disease, Liver Dysfunction | **ACSS3**, AGPAT3, **ATP8A1**, **C2CD2L**, Ca2, **CMC1**, **CNST**, creatine, CREB3, **EMC6**, EMC9, Endothelin, ENTPD2, F2, FAIM2, FIG4, GIPR, GJA1, HNF4A, HSPA8, HTT, **MECR**, **MFAP4**, **MIGA2**, **MYH1**, PEX11B, phosphocreatine, PIK3C3, **PVALB**, **SAA1**, SLC12A3, **SLC22A3**, **SLC39A13**, TM4SF4, VEGFD |
| 10 | 20 | 13 | Dermatological Diseases and Conditions, Organ Morphology, Organismal Injury and Abnormalities | Ap1, **AQP7**, **Bst2**, Cytokeratin, estrogen receptor, hexokinase, **HK2**, **HK3**, Iga, IgG, IgG1, IgG2a, Igm, IL1, IL12 (complex), IL12 (family), Immunoglobulin, Interferon alpha, **ITGB2**, Keratin, **Krt10**, **KRT222**, **KRT7**, **KRT79**, Ldh (complex), MAP2K1/2, MHC Class Ⅱ (complex), **MTCH2**, p85 (pik3r), PI3K (complex), Rxr, **SCGB1A1**, SRC (family), **TCIRG1**, **UCP3** |
| 11 | 16 | 11 | Cellular Assembly and Organization, Cellular Development, Cellular Growth and Proliferation | Alp, **AMACR**, AURKC, CD3, chemokine, **CIRBP**, cytokine, endocannabinoid, **GPAT4**, Histone h3, **HNRNPAO**, Insulin, L-leucine, **LGALS3BP**, LIPA, MAATS1, **MYLPF**, **Ngp**, P38 MAPK, PAIP2B, Pka, Pka catalytic subunit, **PRKAB1**, Pro-inflammatory Cytokine, Proinsulin, Rac, **RALGAPA2**, Ras, Ras homolog, Rb, **RBM3**, **TAF15**, Tnp2, UDP-D-glucose, vitamin K1 |
| 12 | 2 | 1 | Cellular Development, Cellular Growth and Proliferation, Hematological System Development and Function | **BTNL9**, STAT1 |
| 13 | 2 | 1 | Cancer, Connective Tissue Disorders, Developmental Disorder | **Hsdl2**, POR |
| 14 | 2 | 1 | Amino Acid Metabolism, Energy Production, Post-Translational Modification | FITM2, RNF130, **TSPAN18** |
| 15 | 2 | 1 | Cancer, Gastrointestinal Disease, Organismal Injury and Abnormalities | CH-OH group:NAD or NADP oxidoreductase, **DHRS11**, HSD17B |

*Note*: Molecules in bold fonts represent the “Focus Molecules”, which were quantified by TMT.
