# Supplementary material for: Improvement of Endothelial Dysfunction of Berberine in Atherosclerotic Mice and Mechanism Exploring through TMT-Based Proteomics
Source: Oxid Med Cell Longev. 2020 May 31;2020:8683404. doi: 10.1155/2020/8683404 (PMC7284929; doi:10.1155/2020/8683404)
Supplement: Supplementary 5 — Supplementary Figure 1: effects of BBR on cardiac function. Male wild-type (WT) C57BL/6 mice were fed with normal chow diet, and ApoE−/− mice were fed with western-type diet in the presence and absence of berberine (BBR, 78 and 156 mg·kg−1) or the presence of atorvastatin (ATO). After 12 weeks of administration, echocardiography was performed. (a) Representative echocardiograms (M-mode) showing the wall motion. (b) Peak Vel echocardiograms on Doppler of the left ventricular outflow tract. (c) Echocardiographic measurements of ejection fraction (EF), fractional shortening (FS), peak Vel and peak pressure of left ventricular outflow tract in different groups. Data are shown as mean ± SEM. #P < 0.05 versus WT, ∗P < 0.05 versus ApoE−/−. Supplementary Figure 2: disease and function analysis of common differentially expressed proteins according to IPA. The differentially expressed proteins were grouped into three categories: diseases and disorders (a), molecular and cell functions (b), and physiological system development and function (c). Supplementary Figure 3: the molecular profiles of top 4 categories in metabolic disease and cardiovascular disease identified by IPA. (a) The molecular profiles of disorder lipid metabolism, fatty acid oxidation disorder, enzymopathy, and hepatic steatosis in metabolic disease. (b) The molecular profiles of coronary disease, abnormal morphology of the heart, acute coronary syndrome, and occlusion of blood vessel in cardiovascular disease. [file 8683404.f5.docx]

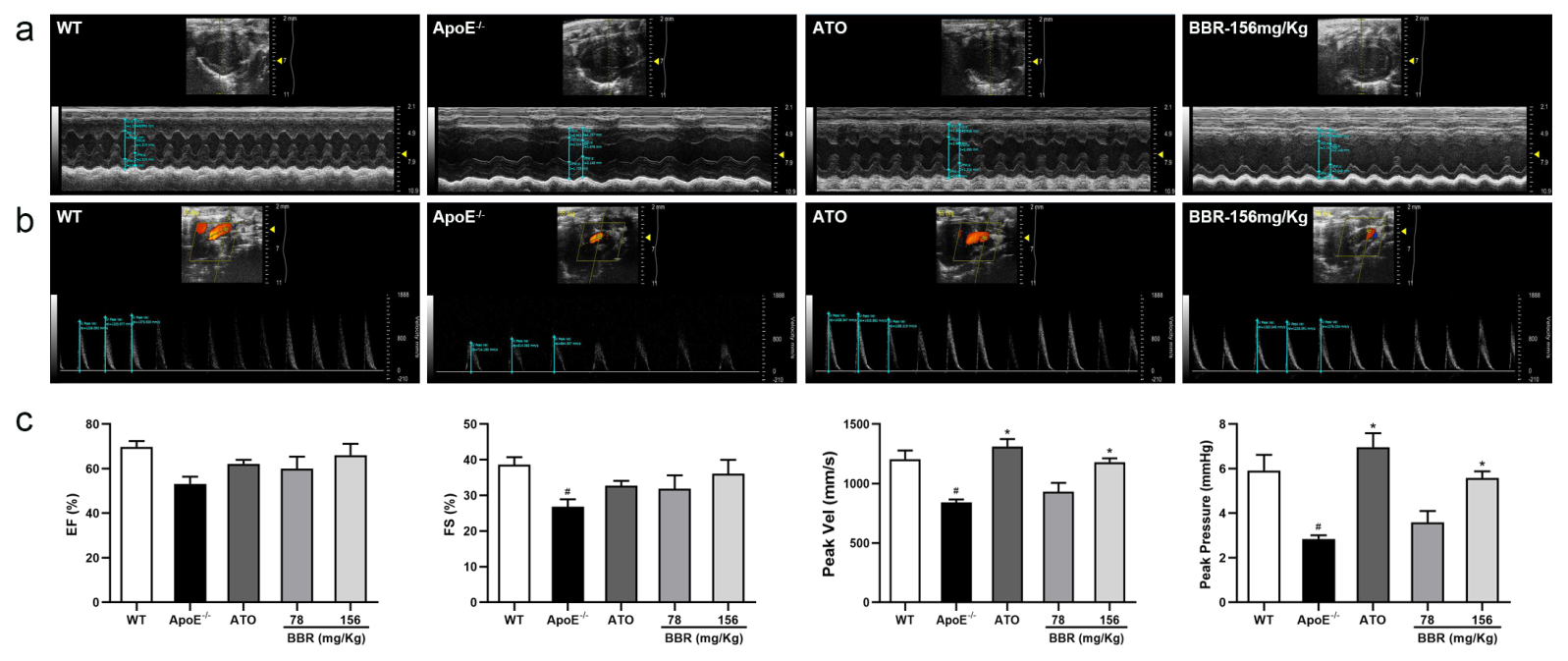


**Supplementary Figure 1. Effects of BBR on cardiac function.** Male wild-type (WT) C57BL/6 mice mice were fed with normal chow diet and ApoE^-/-^ mice were fed with western-type diet in the presence and absence of berberine (BBR, 78, 156 mg·kg^-1^) or the presence of atorvastatin (ATO). After 12 weeks of administration, echocardiography was performed. (a) Representative echocardiograms (M-mode) showing the wall motion. (b) Peak Vel echocardiograms on Doppler of the left ventricular outflow tract. (c) Echocardiographic measurements of ejection fraction (EF), fractional shortening (FS), peak Vel and peak pressure of left ventricular outflow tract in different groups. Data are shown as mean ± SEM. ^#^*P* < 0.05 versus WT, ^*^*P* < 0.05 versus ApoE^-/-^.


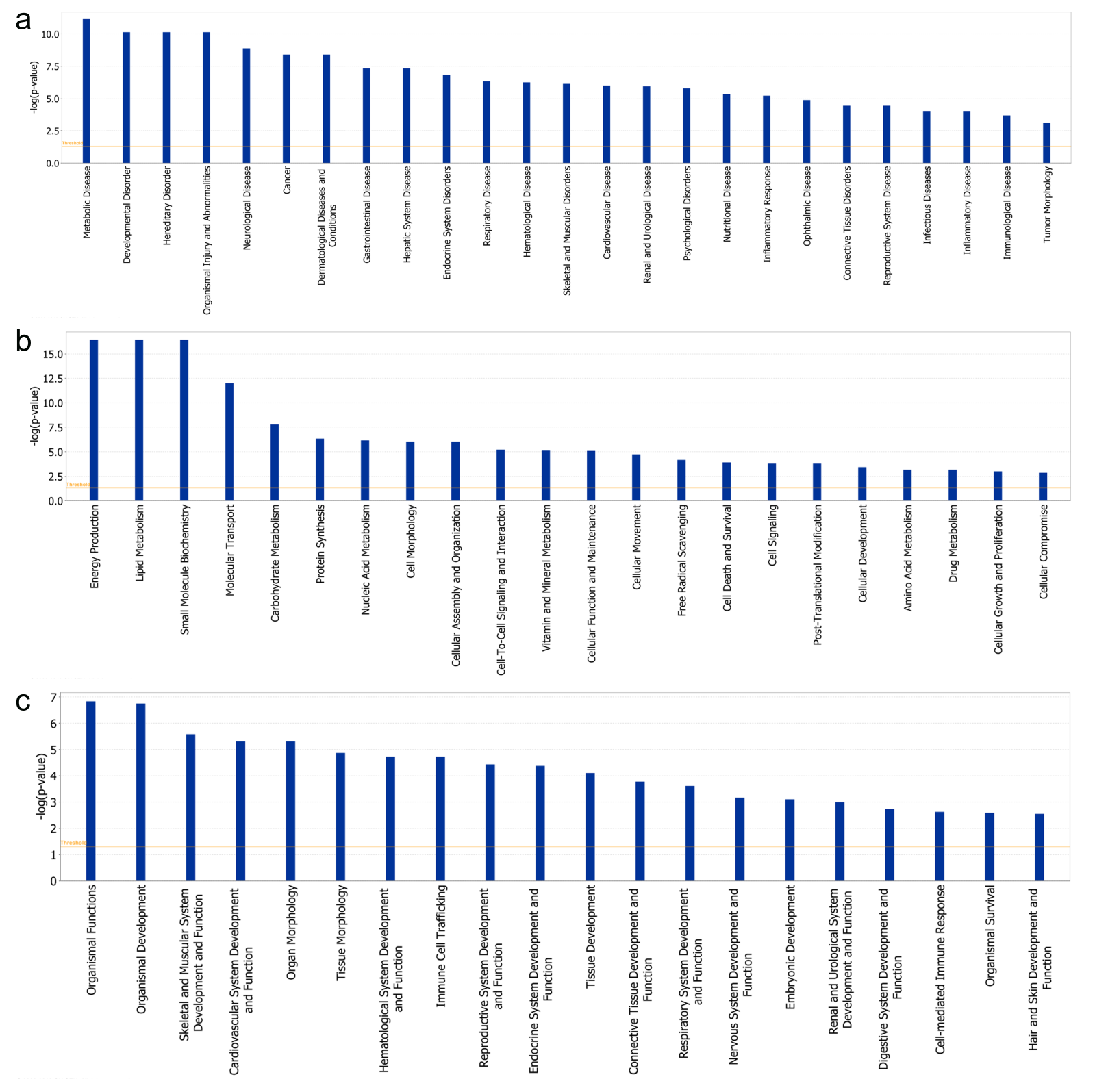


**Supplementary Figure 2. Diseases and functions analysis of common differentially expressed proteins according to IPA.** The differentially expressed proteins were grouped into three categories: Diseases and Disorders (a), Molecular and Cell Functions (b) and Physiological System Development and Function (c).


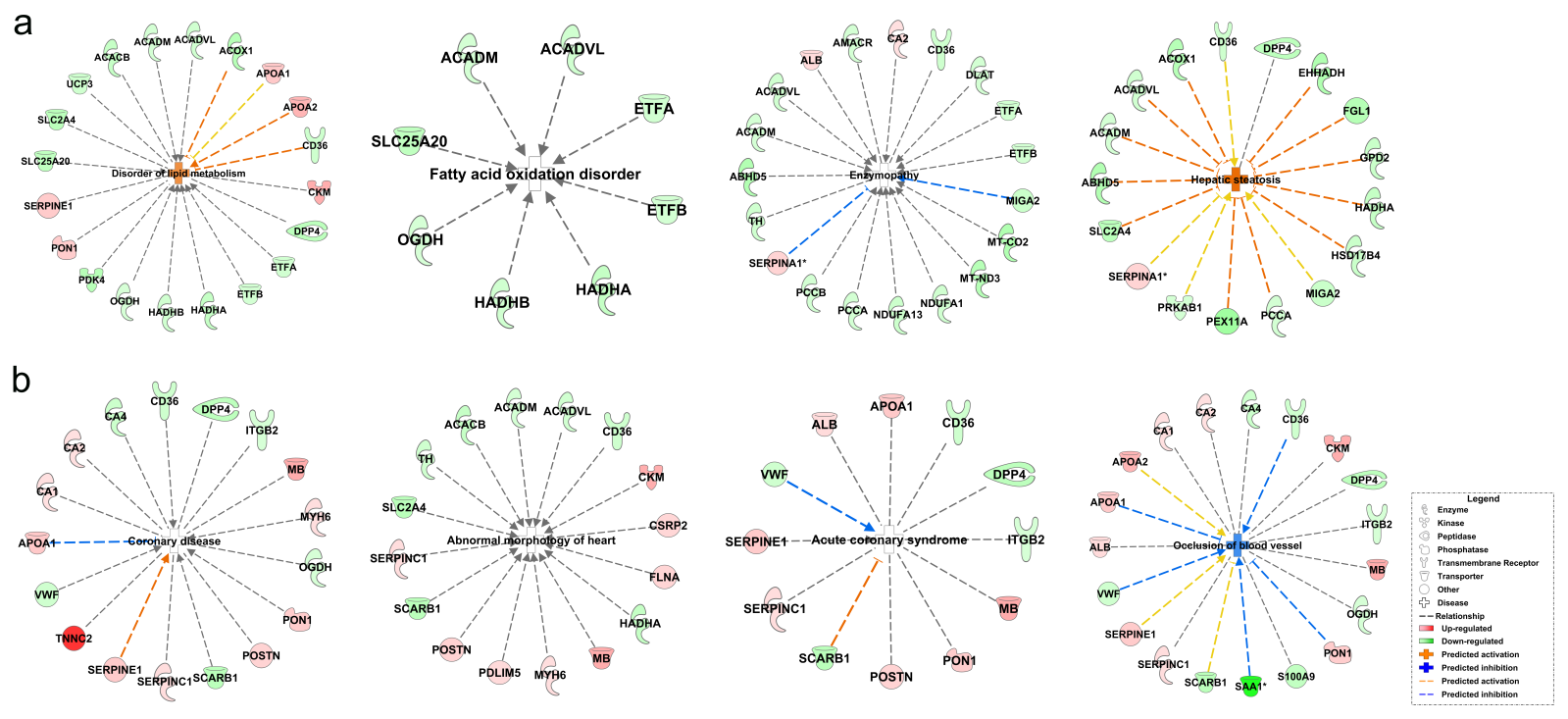


**Supplementary Figure 3. The molecular profiles of TOP 4 categories in metabolic disease and cardiovascular disease identified by IPA.** (a) The molecular profiles of disorder of lipid metabolism, fatty acid oxidation disorder, enzymopathy and hepatic steatosis in metabolic disease. (b) The molecular profiles of coronary disease, abnormal morphology of heart, acute coronary syndrome and occlusion of blood vessel in cardiovascular disease.
